# A New Dicoumarinyl Ether from the Roots of *Stellera chamaejasme* L

**DOI:** 10.3390/molecules19021603

**Published:** 2014-01-27

**Authors:** Jie Li, Qian Shen, Chen-Hao Bao, Li-Ting Chen, Xiang-Rong Li

**Affiliations:** School of Medicine, Zhejiang University City College, No. 48, Huzhou Road, Hangzhou 310015, Zhejiang, China

**Keywords:** *Stellera chamaejasme* L, dicoumarinyl ether, 3-hydroxy-6-methoxy-7,7′-dicoumarinyl ether

## Abstract

A new dicoumarinyl ether, 3-hydroxy-6-methoxy-7,7'-dicoumarinyl ether (**1**), was isolated from the roots of *Stellera chamaejasme* L, together with the known compound umbelliferone (**2**). Their structures were determined on the basis of spectroscopic techniques, including IR, NMR, and HR-ESI-MS.

## 1. Introduction

**Figure 1:**
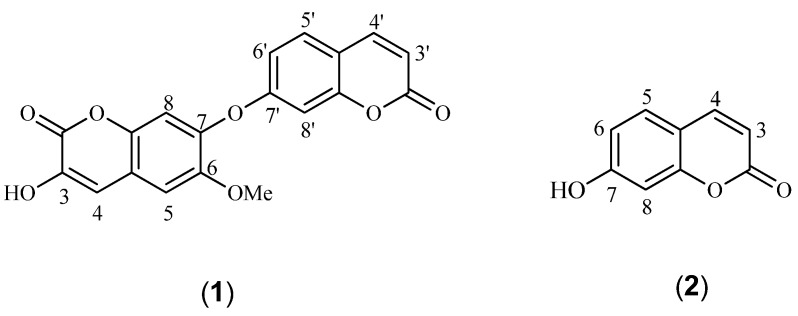
Chemical Structures of **1**–**2**.

*Stellera chamaejasme* L. (*Ruixianglangdu* in Chinese, family Thymelaeaceae), has been used in China as an important traditional medicine for the treatment of scabies, tinea, stubborn skin ulcers, chronic tracheitis, cancer, and tuberculosis [1,2]. Previous phytochemical studies of *S*. *chamaejasme* showed the presence of groups of biflavonoids, diterpenes, lignans, and coumarins [3,4,5,6,7,8,9,10,11,12]. In our continuous studies on the chemical constituents of *S.* *chamaejasme*, a new bicoumarin, 3-hydroxy-6-methoxy-7,7'-dicoumarinyl ether (**1**, Figure 1) was isolated, together with the known coumarin umbelliferone (**2**). Here, we report the isolation and structure elucidation of the new compound.

## 2. Results and Discussion

**Figure 2:**
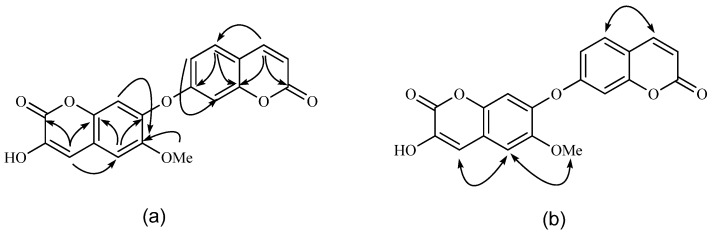
Key HMBC (a), and key NOESY (b) correlations of compound **1**.

Compound **1** was obtained as a white, amorphous powder. HR-ESI-MS (negative) indicated a molecular formula of C_19_H_12_O_7_ by a pseudo molecular ion peak at *m/z* 351.0505 [M−H]^−^. The IR spectrum of **1** displayed absorptions of hydroxyl (3,448 cm^−1^), α,β-unsaturated lactone (1,724 cm^−1^), and phenyl (1,614, 1,580, and 1,456 cm^−1^) functionalities. In the ^1^H-NMR spectrum, vicinal pairs of signals typical of the H-3' and H-4' protons of an AX system at *δ*_H_ 6.31 (1H, d, *J* = 9.6 Hz, H-3') and 7.96 (1H, d, *J* = 9.6 Hz, H-4'), were observed, along with an ABX coupling pattern (*δ*_H_ 7.68 (1H, d, *J* = 8.6 Hz, H-5'), 7.10 (1H, dd, *J* = 8.6, 2.0 Hz, H-6') and 7.06 (1H, d, *J* = 2.0 Hz, H-8')), suggesting the presence of an 7-oxygenated coumarin moiety, which was further confirmed by the NOESY correlation of H-4' and H-5' (Figure 2). 

The other unit was deduced to be a 3-hydroxy-6-methoxy-7-substituted- coumarin as follows: H-4 (*δ*_H_ 7.80) and H-5 (*δ*_H_ 7.24) showed a correlation in the NOESY experiment. Accordingly, cross-peaks between H-4 and C-5 (*δ*_C_ 109.9) and between H-5 and C-4 (*δ*_C_ 131.5) were evident in the HMBC experiment (Table 1). The correlation between the methoxy protons (*δ*_H_ 3.92) and H-5 in the NOESY spectrum indicated that the methoxy group was attached to C-6 (*δ*_C_ 146.6), which was further supported by the long-range correlation between the methoxy protons and C-6 in the HMBC spectrum. Compared with the known compound daphnoretin that was isolated from the same plant [13], the resonance of H-8' in **1** was shifted downfield by 0.22 ppm, and the H-8 one shifted upfield by 0.25 ppm, indicating that the two coumarin moieties were connected at C-7 and C-7' via an oxygen bridge, which was further supported by ESIMS/MS experiment of **1**. The daughter ion peaks at *m/z* 206 and *m/z* 144 were derived from fission of the C-7'−O bond. Therefore, compound **1** was elucidated as 3-hydroxy-6-methoxy-7,7'-dicoumarinyl ether and named neodaphnoretin. The known compound was identified as umbelliferone (**2**) by comparison of its ^1^H- and ^13^C-NMR and MS data with published data.

**Table 1:** NMR data of neodaphnoretin (**1**) in CD_3_COCD_3_ (600 MHz for ^1^H, 150 MHz for ^13^C).

NO.	δ_H_ Mult (*J* = Hz)	δ_C_	HMBC	NO.	δ_H_ Mult (*J* = Hz)	δ_C_	HMBC
2		157.8 s		3'	6.31 d (9.6)	115.0 d	2', 10'
3		137.5 s		4'	7.96 d (9.6)	144.4 d	2', 5', 9'
4	7.80 s	131.5 d	2, 3, 5, 9	5'	7.68 d (8.6)	130.6 d	4', 7', 9'
5	7.24 s	109.9 d	4, 6, 7, 9	6'	7.10 dd (8.6, 2.0)	114.3 d	8', 10'
6		146.6 s		7'		161.1 s	
7		151.2 s		8'	7.06 d (2.0)	104.9 d	6', 7'
8	6.90 s	103.7 d	6, 7, 9, 10	9'		156.5 s	10'
9		149.0 s		10'		115.5 s	
10		111.6 s		6-OCH_3_		56.7 q	
2'		160.7 s			3.92 s		6

## 3. Experimental

### 3.1. General

Melting points were measured on a Thermal Values analytical microscope and are uncorrected. IR spectra were recorded on a FI−IR 200SXY spectrophotometer (Nicolet, Madison, WI, USA). The high resolution-electrospray ionization-mass spectra (HR-ESI-MS) were acquired with a Micromass Q−TOF mass spectrometer (Waters Corporation, Carlsbad, CA, USA). NMR spectra were measured (^1^H at 600 MHz and ^13^C at 150 MHz) with TMS as the internal standard on a DD2 600 NMR instrument (Agilent, Santa Clara, CA, USA). Silica gel G_254_ and H (Qingdao Sea Chemical Factory, Qingdao, China) were used for TLC and column chromatography, respectively.

### 3.2. Plant Material

The roots of *S.* *chamaejasme* were collected in Kunming, Yunnan Province, China, in October, 2010. The plant was identified by Le Cai (Yunnan University). A voucher specimen (LJ-RXLD1008) was deposited with the Zhejiang University City College.

### 3.3. Extraction and Isolation

Air-dried powdered roots (3.0 kg) of *S. chamaejasme* were extracted exhaustively with 95% aq. EtOH (2 L) at r.t. for four times (7 days for each time). After concentration *in vacuo*, a crude extract (360 g) was obtained, which was suspended in H_2_O (1 L), and the suspension was extracted three times successively with petroleum ether, EtOAc and BuOH (1 L). The EtOAc fraction (160 g) was subjected to column chromatography (CC) with a PE/EtOAc gradient system of increasing polarity (9:1, 8:2, 7:3, 6:4, 5:5) as eluent to give six fractions (Fr. 1–6). Fr. 3 was rechromatographed on a SiO_2_ column with CHCl_3_/MeOH (98:2→95:5) to give compounds **1** (34 mg) and **2** (23 mg).

*Neodaphnoretin* (**1**) was obtained as white, amorphous powder, mp 133–135 °C, from CHCl_3_−MeOH (97:3). IR (KBr) cm^−1^: 3,448, 1,724, 1,614, 1,580, 1,456; ^1^H-NMR (CD_3_COCD_3_) *δ*: see Table 1; ^13^C-NMR (CD_3_COCD_3_) *δ*: see Table 1; HR-ESI-MS: *m/z* [M−H]^−^ 351.0505 (calcd. for C_19_H_11_O_7_, 351.0505).

*Umbelliferone* (**2**) was obtained as colorless crystals, mp 222–224 °C, from MeOH; ^1^H-NMR (CD_3_OD) *δ*: 7.83 (1H, d, *J* = 9.4 Hz), 7.44 (1H, d, *J* = 8.5 Hz), 6.79 (1H, dd, *J* = 8.5, 2.0 Hz), 6.70 (1H, d, *J* = 2.0 Hz), 6.17 (1H, d, *J* =9.4 Hz); ^13^C-NMR (CD_3_OD) *δ*: 162.3, 161.8, 155.8, 144.6, 129.2, 113.1, 111.6, 102.0.

## 4. Conclusions

A new dicoumarinyl ether, 3-hydroxy-6-methoxy-7,7'-dicoumarinyl ether (**1**) was isolated from the EtOH extract of the roots of *Stellera chamaejasme* L together with the known compound umbelliferone (**2**).

## Supplementary Materials

Supplementary materials can be accessed at: http://www.mdpi.com/1420-3049/19/2/1603/s1.
