# Supplementary material for: A New Dicoumarinyl Ether from the Roots of Stellera chamaejasme L
Source: Molecules. 2014 Jan 27;19(2):1603–7. doi: 10.3390/molecules19021603 (PMC6271840; doi:10.3390/molecules19021603)
Supplement: Supplementary file 1 [file molecules-19-01603-s001.pdf]

Supplementary Materials

Figure S1. ^{1}H-NMR spectrum of compound **1** at 600 MHz in CD_3COCD_3.

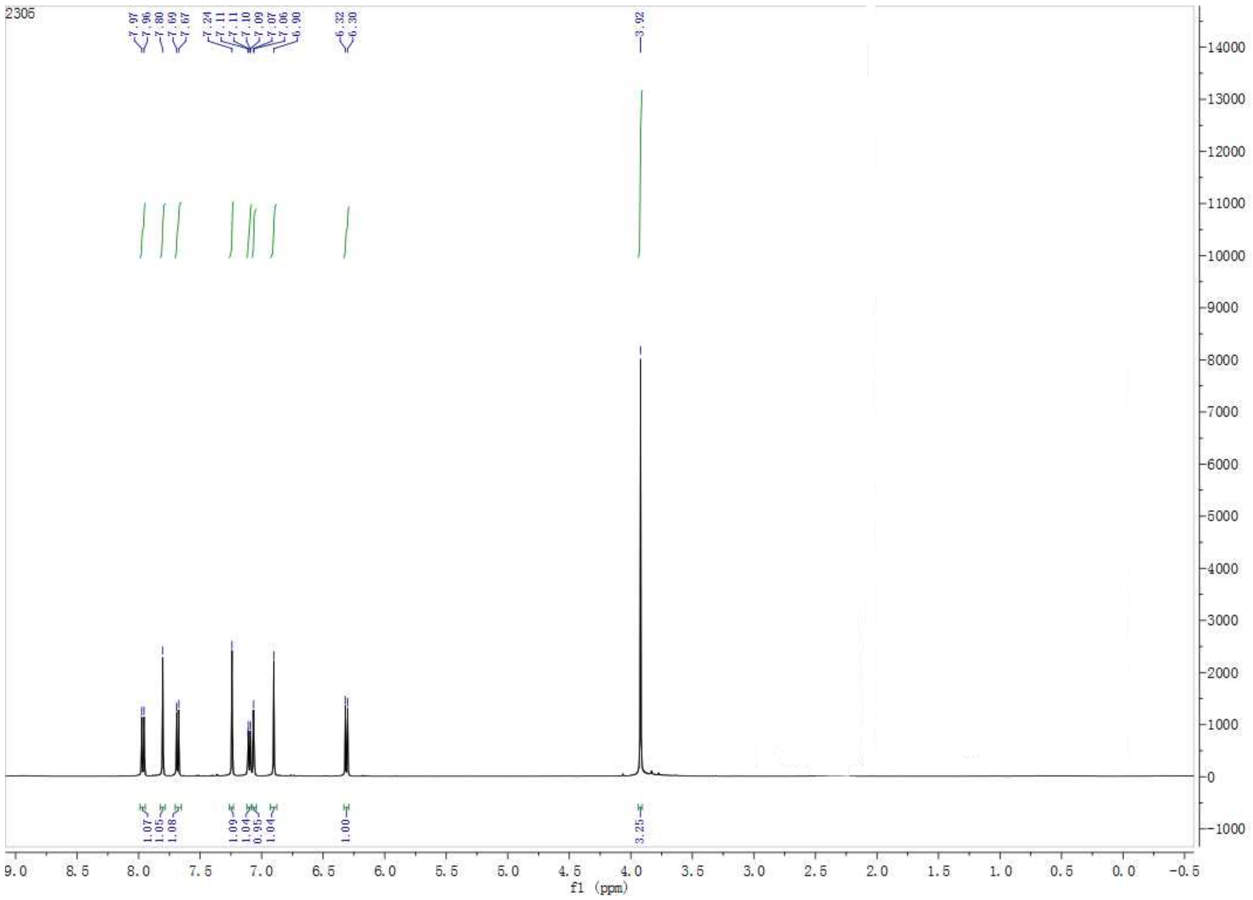

Figure S2. ^{13}C-NMR spectrum of compound **1** at 150 MHz in CD_3COCD_3.

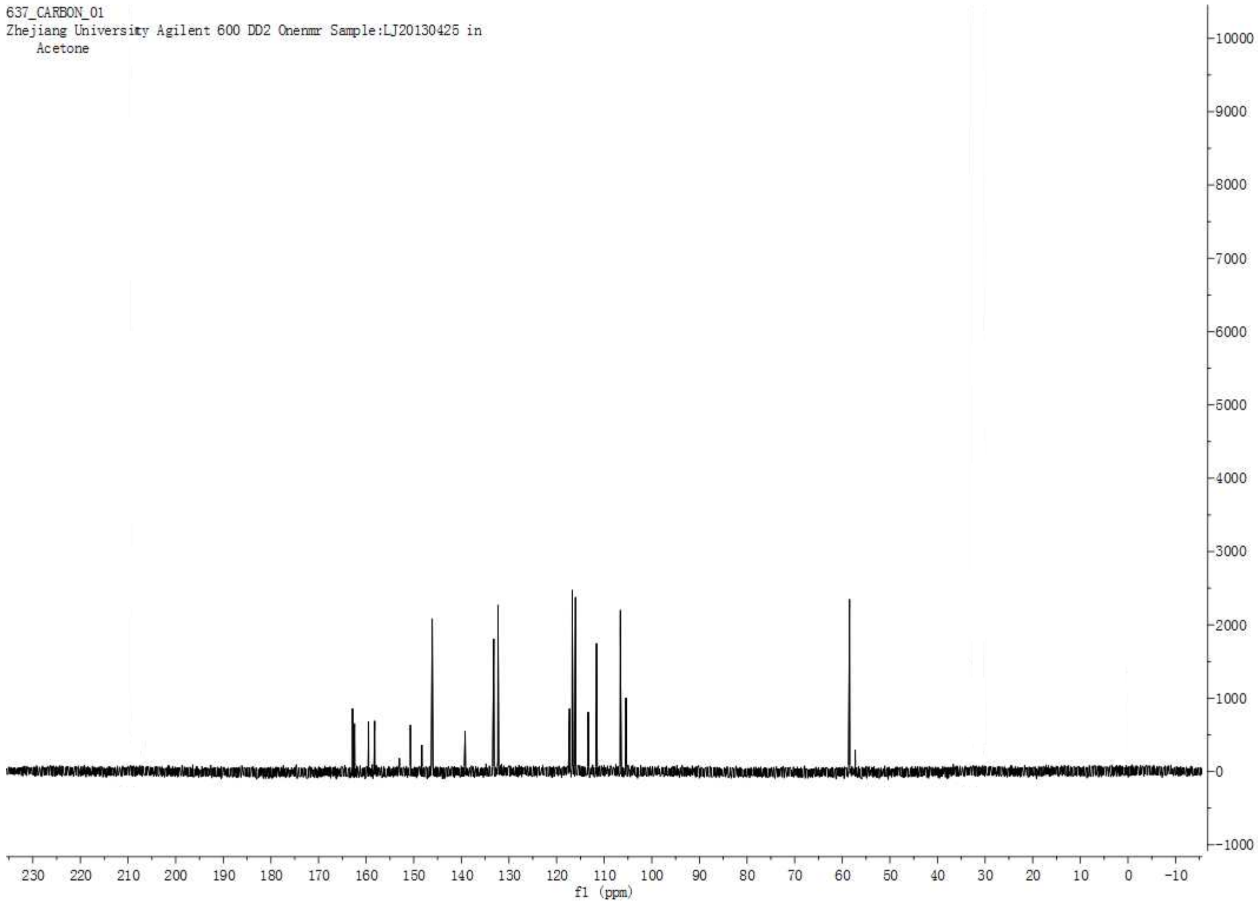

Figure S3. ^1H-^1H COSY spectrum of compound **1** at 600 MHz in CD_3COCD_3.

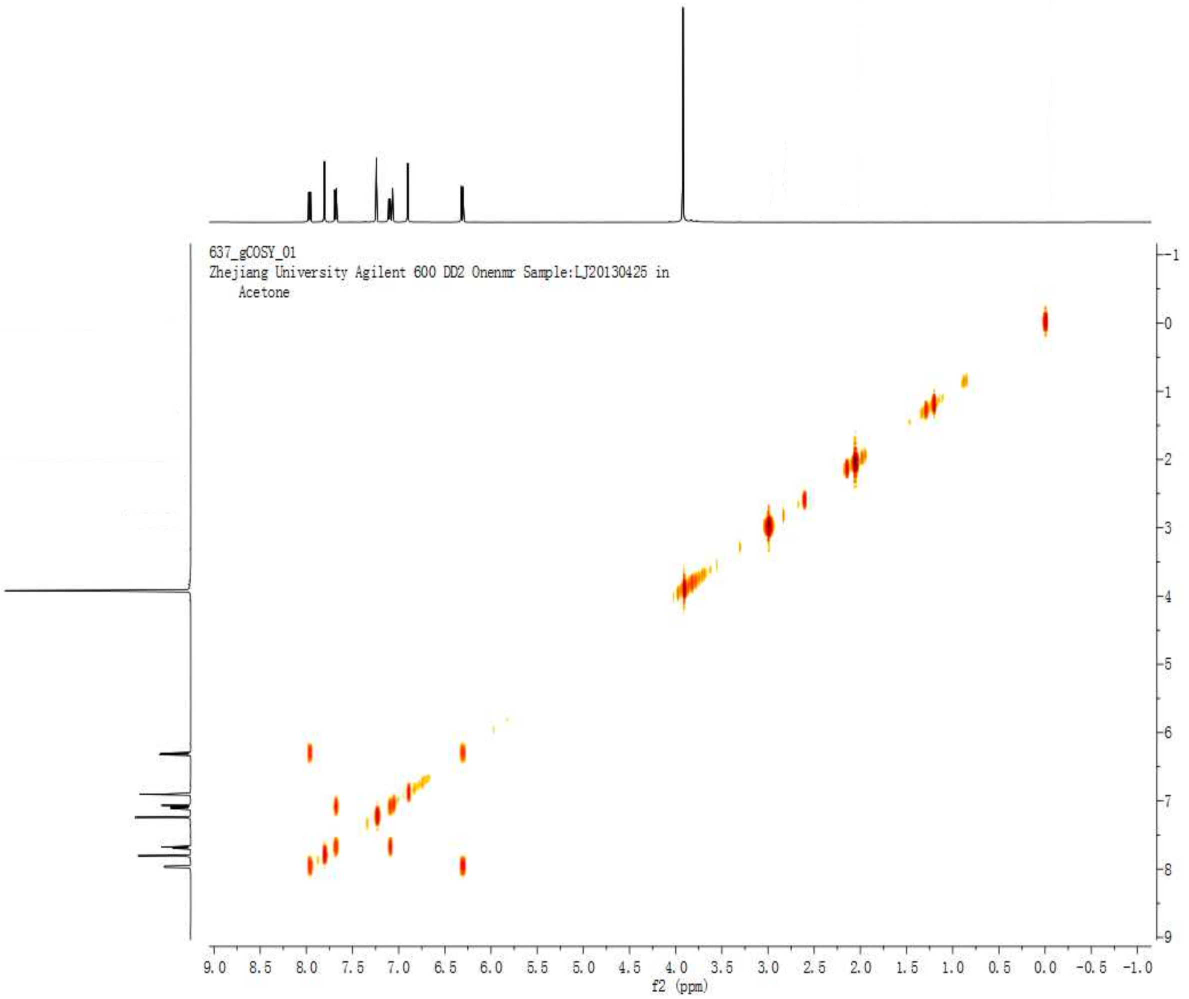

Figure S4. HSQC spectrum of compound **1** at 600 MHz in CD_3COCD_3.

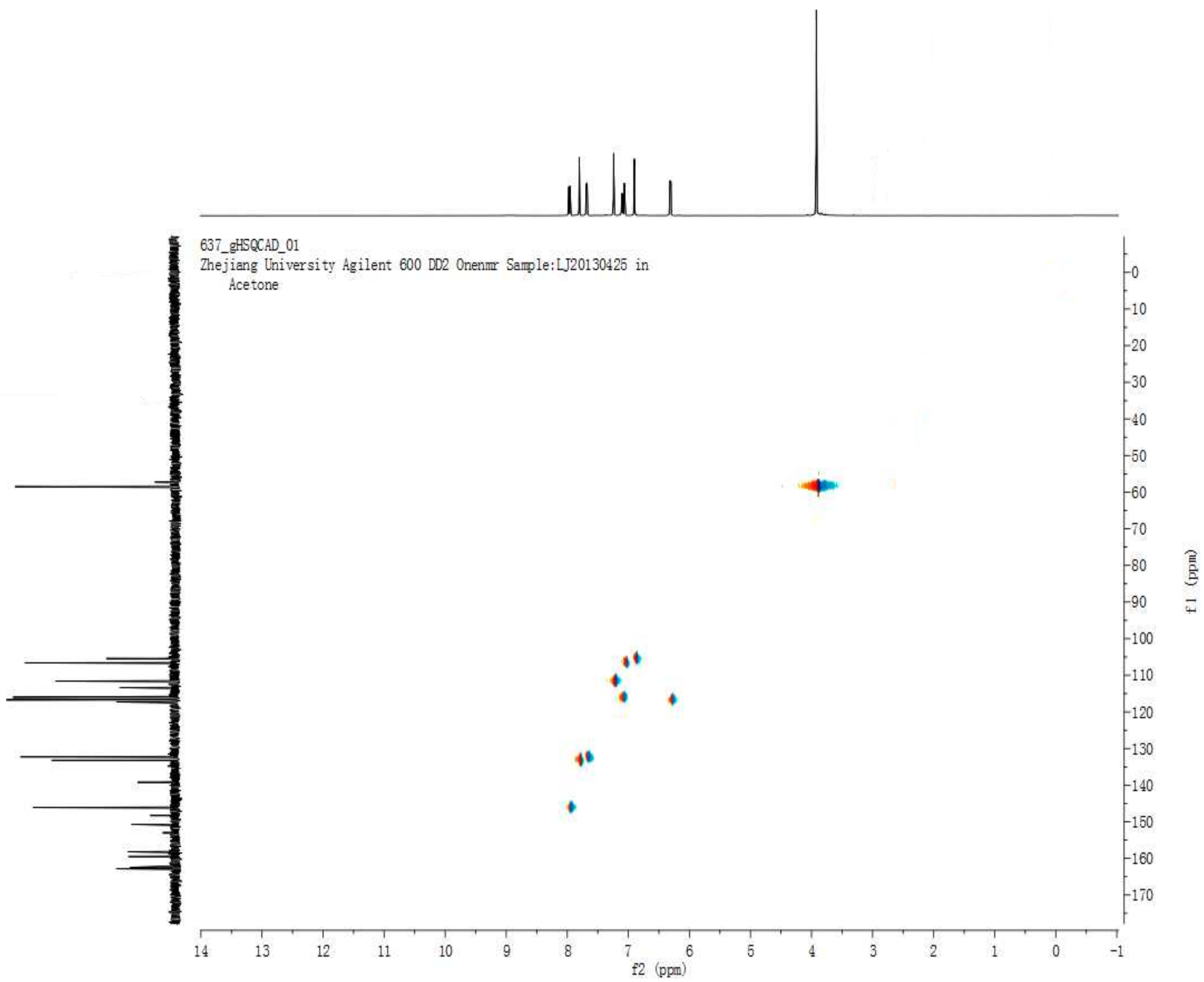

Figure S5. HMBC spectrum of compound **1** at 600 MHz in CD_3COCD_3.

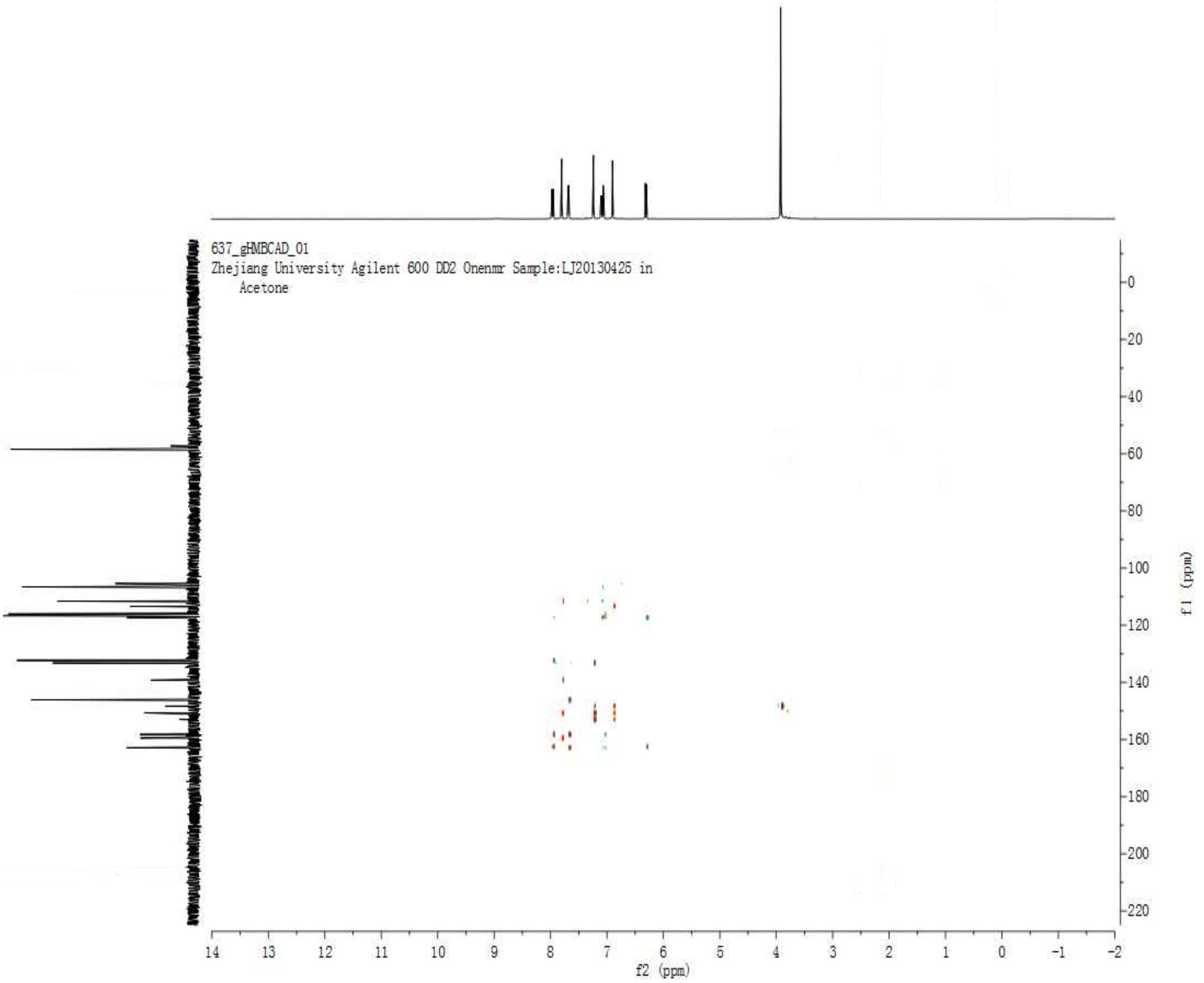

Figure S6. HMBC spectrum of compound **1** at 600 MHz in CD_3COCD_3.

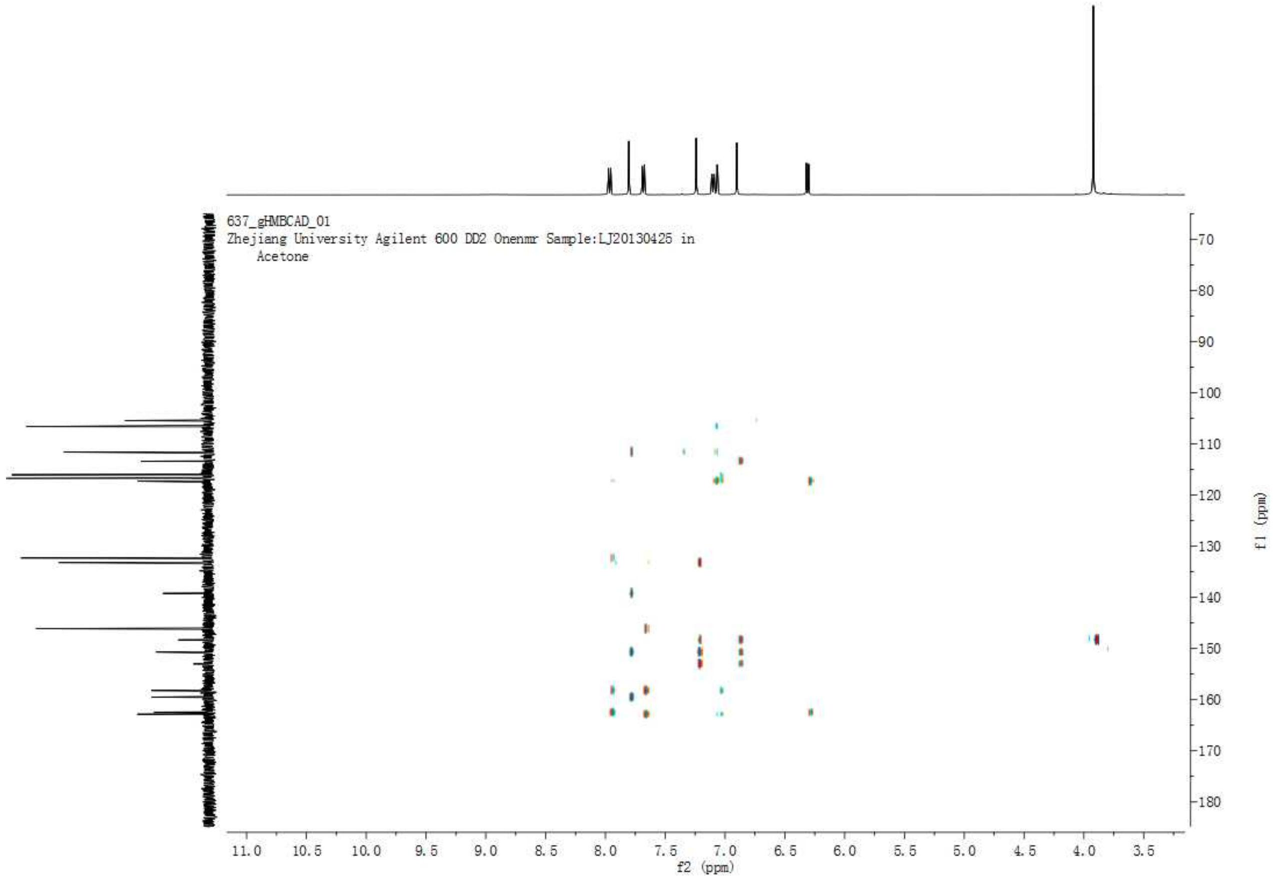

Figure S7. NOESY spectrum of compound **1** at 600 MHz in CD_3COCD_3.

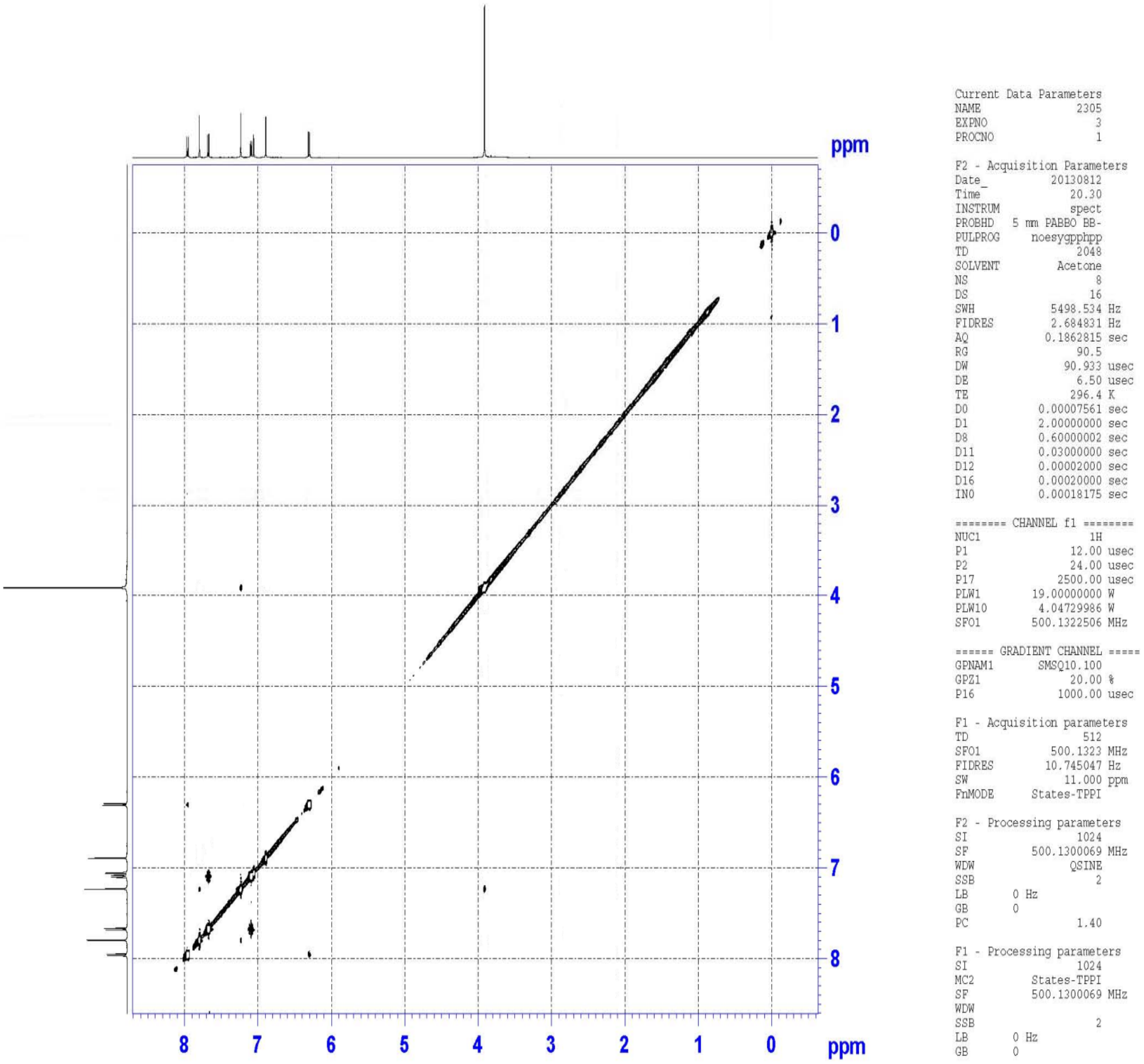

Figure S8. NOESY spectrum of compound **1** at 600 MHz in CD_3COCD_3.

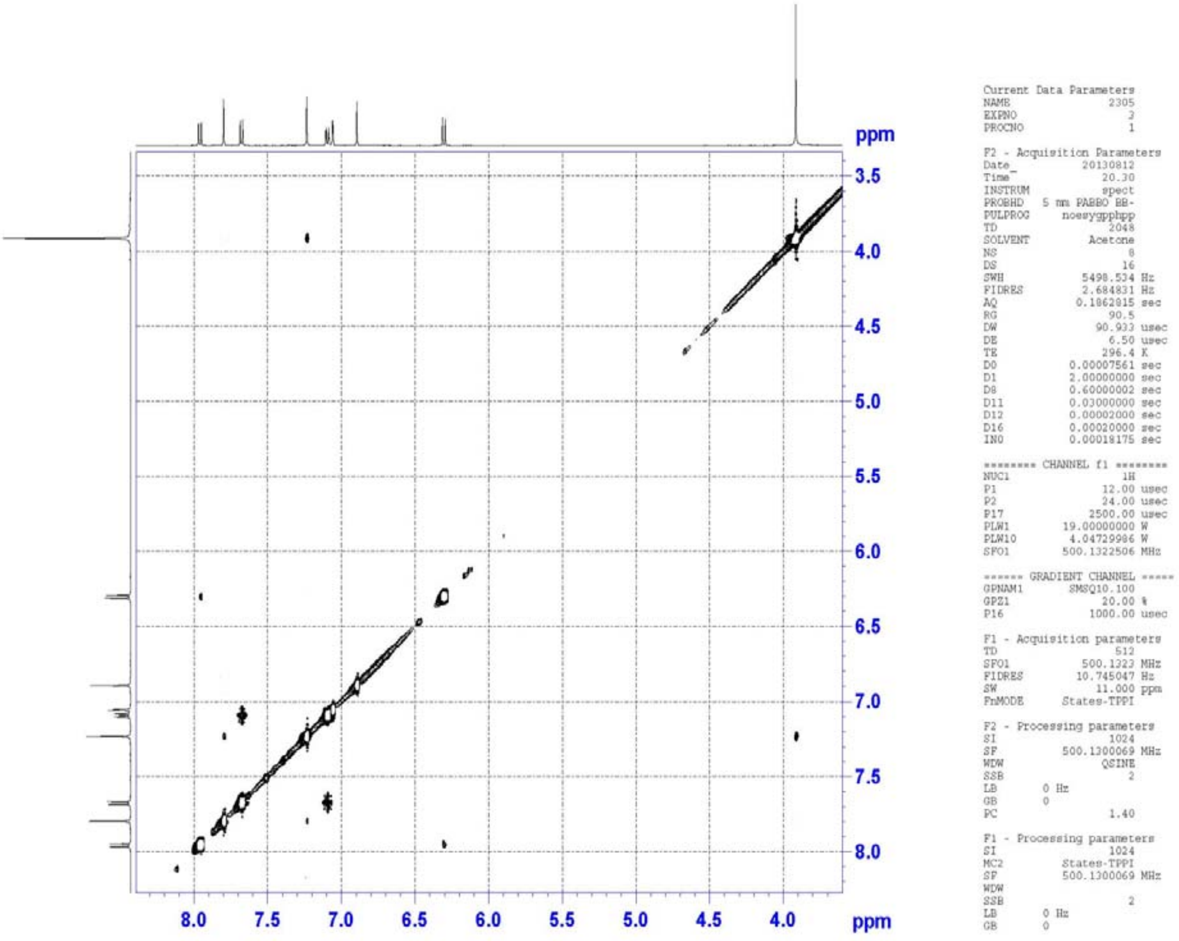

Figure S9. HR-ESI-MS spectrum of compound **1** in MeOH.

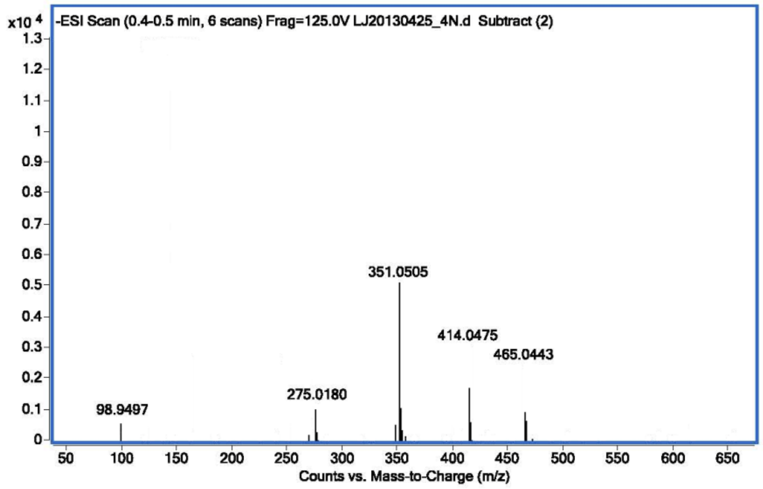

Ion Formula	m/z	Calc m/z	Diff (ppm)	DBE
C19 H11 O7	351.0505	351.0505	-0.06	14.5
